# Predictive biomarkers of COVID-19 prognosis identified in Bangladesh patients and validated in Japanese cohorts

**DOI:** 10.1038/s41598-024-63184-8

**Published:** 2024-06-03

**Authors:** Kazuko Uno, Abu Hasan, Emi E. Nakayama, Rummana Rahim, Hiromasa Harada, Mitsunori Kaneko, Shoji Hashimoto, Toshio Tanaka, Hisatake Matsumoto, Hitoshi Fujimiya, Tatsuo Shioda, Mizanur Rahman, Kazuyuki Yoshizaki

**Affiliations:** 1 IFN and Host-Defense Research Laboratory, Louis Pasteur Center for Medical Research, Kyoto, Kyoto, 606-8225 Japan; 2 Evercare Hospital Dhaka, Plot-81, Block-E, Bashundhara R/A, Dhaka, 1229 Bangladesh; 3 Research Institute for Microbial Diseases, Osaka University, Suita, Osaka, 565-0781 Japan; 4 Yao Tokushukai General Hospital, Yao, Osaka 581‑001 Japan; 5 Suita Tokushukai Hospital, Suita, Osaka 565‑0814 Japan; 6 Osaka Prefectural Hospital Organization Osaka Habikino Medical Center, Habikino, Osaka 583‑8588 Japan; 7 Kinki Central Hospital, Itami, Hyogo 664-8533 Japan; 8 Trauma and Acute Critical Care Center, Osaka University, Suita, Osaka 565‑0871 Japan; 9 Dynacom Co., Ltd., Chiba, Chiba 261-7125 Japan; 10 Department of Organic Fine Chemicals, Institute of Scientific and Industry Research, Osaka University, Suita, Osaka Japan

**Keywords:** COVID-19 prognosis, COVID-19 biomarker, Lasso method, Bangladesh, Japan, Immunology, Microbiology, Medical research

## Abstract

Despite high vaccination rates globally, countries are still grappling with new COVID infections, and patients diagnosed as mild dying at home during outpatient treatment. Hence, this study aim to identify, then validate, biomarkers that could predict if newly infected COVID-19 patients would subsequently require hospitalization or could recover safely with medication as outpatients. Serum cytokine/chemokine data from 129 COVID-19 patients within 7 days after the onset of symptoms in Bangladesh were used as training data. The majority of patients were infected with the Omicron variant and over 88% were vaccinated. Patients were divided into those with mild symptoms who recovered, and those who deteriorated to moderate or severe illness. Using the Lasso method, 15 predictive markers were identified and used to classify patients into these two groups. The biomarkers were then validated in a cohort of 194 Covid patients in Japan with a predictive accuracy that exceeded 80% for patients infected with Delta and Omicron variants, and 70% for Wuhan and Alpha variants. In an environment of widespread vaccination, these biomarkers could help medical practitioners determine if newly infected COVID-19 patients will improve and can be managed on an out-patient basis, or if they will deteriorate and require hospitalization.

## Introduction

The COVID-19 pandemic has infected over 700 million people and resulted in more than 7 million deaths worldwide as of September 2023. Prior to the availability of vaccines, approximately 80% of infected patients were asymptomatic or mildly ill, while 15%, mainly the elderly, developed severe pneumonia, and 5% progressed to the fatal acute respiratory distress syndrome (ARDS)^1^. Severe COVID-19 has also been associated with various complications, including vasculitis, thrombosis, cerebral infarction, myocardial damage, and multiple organ failure^2,3^. Risk factors for severe COVID-19 symptoms include underlying conditions such as cardiovascular disease, hypertension, diabetes, chronic lung disease, and chronic kidney disease, as well as demographic factors like age, obesity, and smoking^4–7^.

In light of the above, hospitalization rates across the globe was extremely high, leading to shortages in not only hospital beds, but also medical personnel, ventillators and other essential medical equipment. In Japan for example, at the beginning of the pandemic, mortality rate was high and all COVID patients were hospitalized which caused a massive over-run on medical facilities. As a solution, the government mandated that some private hotels were to be used to house infected patients; this lasted until May 2023 well after vaccination had started.

Mortality rates gradually decreased when global vaccination began in early 2021, by end of that year vaccination rates stood at about 80% in Japan and 90% in individuals aged over 50 (https://www.mhlw.go.jp/stf/seisakunitsuite/bunya/kenkou_iryou/kenkou/kekkaku-kansenshou/yobou-sesshu/syukeihou_00002.html). However, the emergence of the Omicron and other variants presented new challenges and in August 2021 Japan had a doubling of infections with the span of a week even in rural areas. The initial Omicron variant, BA.1, showed reduced affinity for lung epithelial cells but higher transmissibility, resulting in attenuated disease severity^8–10^. Subsequent Omicron variants such as BA.5 and XBB regained affinity for the lungs, and the effectiveness of treatments with anti-S protein monoclonal antibodies declined due to escape mutations^11,12^. Antiviral drugs such as Remdesivir, Ritonavir-boosted Nirmatrelvir and Molnupiravir^13^ were available but the number of deaths in Japan in 2022 exceeded the number of cases seen in 2020–2021, thereby overwhelming hospital capacity. As a result, due to new infections skyrocketing, even the makeshift hospitals housed inside hotels, exceeded maximum capacity. Hence since early 2022 in Japan, only cases that were judged as being severe were hospitalized, leading to a well-needed steady decrease in the number patients who were admitted. By September 2022, out-pattient treatment had become commonplace.

Outpatient care however presented other major challenges including high death rates among the elderly and among patients who were considered as mild on their first hospital visit. Data from the Japanese Ministry of Health Labour and Welfare in 2022, showed that among patients who died during home care, 58% were 80 years and older, and only 20% were confirmed as being unvaccinated. Additionally, 42% of these patients who died while being treated on an out-patient basis were initially diagnosed as mild cases (https://www.mhlw.go.jp/content/10900000/001021500.pdf).

In this context where COVID patients can still experience severe disease progression despite the widespread use of vaccines, having a predictive model that can easily can distinguish patients at high risk of deterioration with just a blood sample would be highly beneficial.

Patients with severe COVID-19 have exhibited elevated levels of inflammatory cytokines and chemokines^4,5,7,14,15^, especially Interleukin 1 (IL-1), Tumor Necrosis Factor alpha (TNFα), and IL-6^16–19^. This has led to the hypothesis that ARDS in COVID-19 is driven by a cytokine storm^20,21^. With this in mind, in this study, we quantified cytokines/chemokines/soluble receptors in serum samples from COVID-19 patients in Bangladesh who visited the hospital during the omicron stage within 7 days on the onset of symptoms. We used machine learning techniques to successfully identify a combination of 15 biomarkers that could predict if patients would subsequently deteriorate to severe COVID-19 or if they would have only mild symptoms that could be treated with out-patient care.

## Results

### Predicting patients’ outcome using pretreatment serum cytokine/chemokine levels within 7 days of onset of COVID-19 from Bangladesh data

The serum from patients in Bangladesh collected December 2021–December 2022 was used to train this decision model for several reasons (Table 1). First, the Japanese serum of COVID patients covers a period of approximately 2 years, during which several COVID-19 variants (Wuhan, Alpha, Delta, and Omicron) emerged, raising concern about the model’s reliability due to the diversity of the data. In contrast, the Bangladesh data was limited to a single strain, and serum sample were collected immediately after the onset of the symptoms as described in the Materials and Methods (Table 2). For these reasons, the Bangladeshi samples were more likely to satisfy the uniformity required for the learning model, and therefore the biomarkers idenitified in the Bangladeshi sample were used to predict recovery or deterioration in Japanese patients.

**Table 1:** Demographic of COVID-19 patients.

Demographic data of COVID-19 patients				
Characteristics	Mild	Moderate	Severe	Total
Bangladesh (n)	64	46	19	129
Age--median year (IQR)	47 (27–59)	68 (55–76)	75 (60–80)	59 (36–73)
Sex (Male: Female)	43:21	21:25	12:7	76:53
Japan (n)	51	25	121	197
Age-median year (IQR)	64 (17–99)	73 (20–93)	75(19–97)	73(17–99)
Sex (Male: Female)	22:29	16:9	85:36	123:74

**Table 2:** Timing of sampling of patient specimens.

Period	Bangladesh	Japan				
	2021/12/25–2022/9/21	2020/6/14–2020/10/9	2020/10/10–2021/2/28	2021/3/1–2021/6/20	2021/6/21–2021/10/17	2021/12/26–2022/6/19
Wave in Japan		2nd wave	3rd wave	4th wave	5th wave	6th wave
Virus main variant	Omicron	Wuhan	Wuhan	Alpha	Delta	Omicron
Final outcome						
Mild: n (%)	64 (49.6)	3 (15.8)	19 (25.7)	11 (25.6)	10 (31.3)	8 (27.6)
Moderate: n (%)	46 (35.7)	1 (5.3)	10 (13.5)	5 (11.6)	6 (18.8)	3 (10.3)
Severe: n (%)	19 (14.7)	15 (78.9)	45 (60.8)	27 (62.8)	16 (50)	18 (62.1)
Total: n	129	19	74	43	32	29

**Figure 1:**
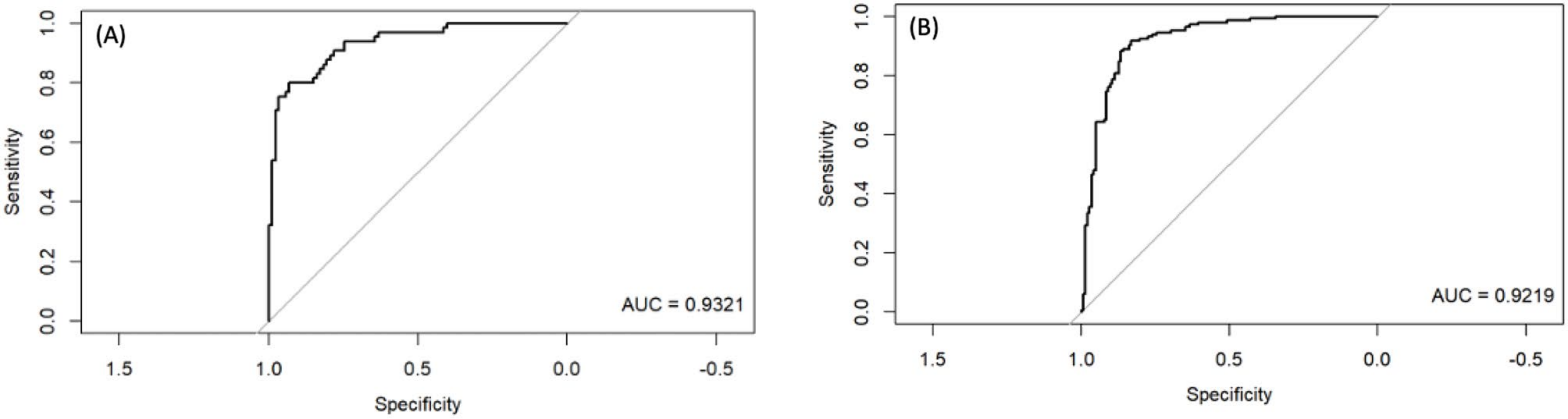
Comparison of ROC curves generated using Bangladeshi and Japanese data. (**A**) ROC curves obtained by evaluating the performance of the logistic regression model using the Bangladeshi training data. (**B**) ROC curves resulting from applying the same logistic regression model to the validation data of the Japanese population.

The prediction model was constructed by categorizing patients in two groups. The first group was labeled “no hospitalization required” because they were only mildly ill (N = 64 patients) throughout the course of the disease and did not develop pneumonia (although some wealthy patients were hospitalized due to anxiety). The second group (N = 65) was labeled “hospitalization required”, because although they were only mildly ill initially, they subsequently developed pneumonia. Additionally, data from 23 healthy individuals were added to the “no hospitalization required” group to ensure that the model could reliably predict that hospitalization is not necessary when the data from healthy subjects were entered. When the number of patients in each group was known, variables were selected using Logistic LASSO regression^22^ with the weight of the inverse of the ratio of the number of each on the objective variable side, and we identified a stable 15-biomarker model with an AUC of 0.9321 (Fig. 1A).

As the number of patients in each group is known, the variables were selected using Logistic LASSO regression^22^ with weights on the objective variable side that are the inverse of the respective headcount ratios, and 15 predictive markers were identified.

Table 3 presents an overview of the performance metrics for this logistic regression model. For the training data (Bangladesh), the model showed a specificity of approximately 91.95%, a sensitivity of 84.85%, and an overall accuracy of 88.89%. Both the negative predictive value (NPV) and the positive predictive value (PPV) were found to be 88.89%. When validated with the Japanese dataset, the results were similar, although the model displayed a lower specificity of about 87.32% and a lower sensitivity of 86.30%. With an accuracy of 86.81%, it was also slightly lower than the training dataset. The NPV was 86.11%, while the PPV were both at 87.50%. These metrics suggest that the logistic regression model performance was consistent across the two diverse datasets, demonstrating robust specificity, sensitivity, and accuracy in both training and validation phases.

**Table 3:** Performance Metrics of the Logistic Regression Model for Both Training and Validation Datasets.

Dataset	Specificity	Sensitivity	Accuracy	NPV	PPV
Bangladesh	0.9195	0.8485	0.8889	0.8889	0.8889
Japan	0.8732	0.863	0.8681	0.8611	0.875

**Figure. 2:**
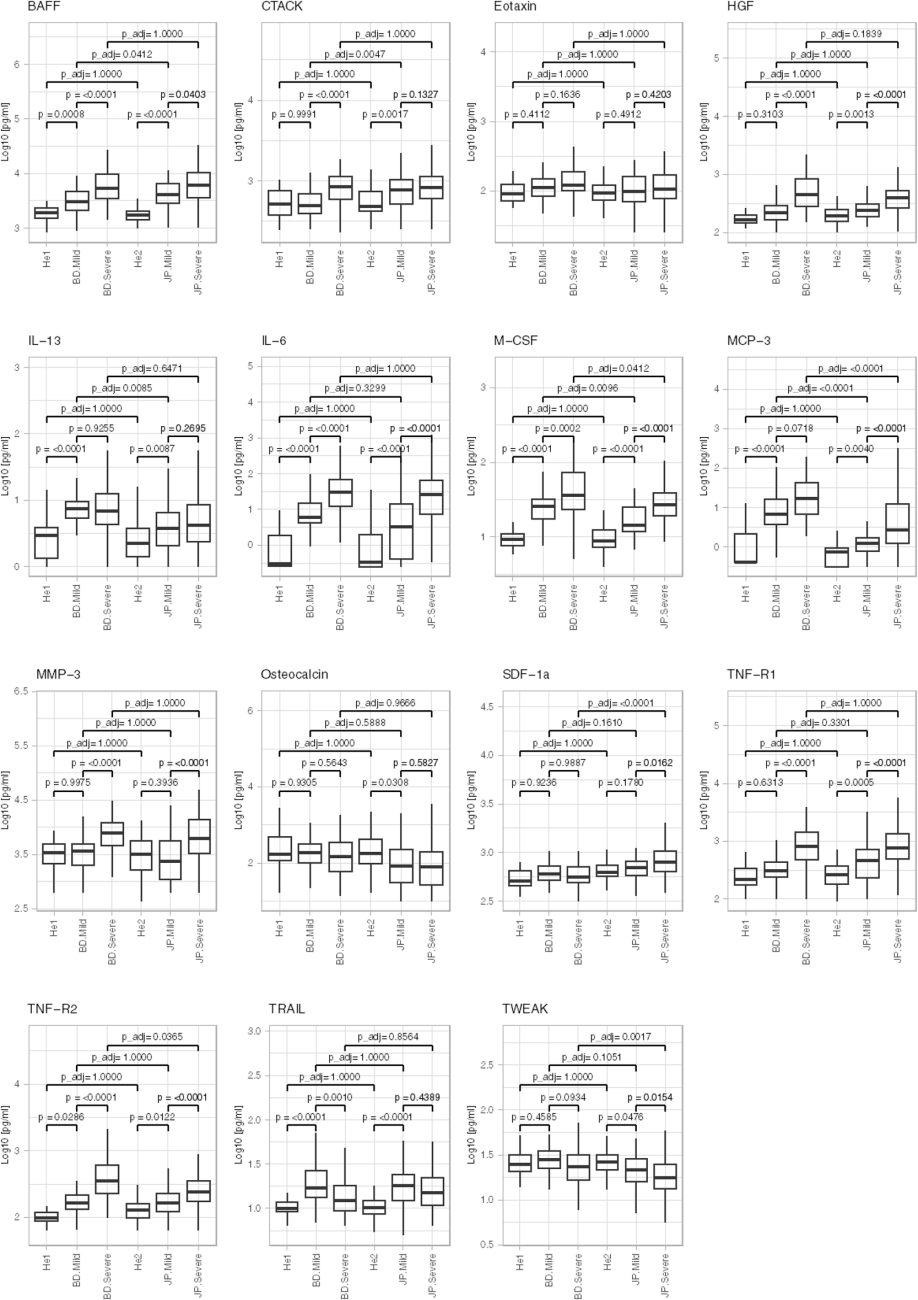
Overview of value of fifteen cytokine/chemokine/soluble receptors in comparison with mild and moderate/severe patients Bangladesh and Japan. Fifteen predictive markers selected from the Bangladesh data are shown. The data are divided into healthy (He1, He2), Bangladesh (BD) and Japanese (JP) mild, and severe cases. The boxplots show medians (middle line) with first and third quartiles (boxes), while the whiskers show 1.5× the interquartile range (IQR) above and below the box.

The 15 predictive biomarkers extracted from the Bangladesh data are: BAFF, CTACK, Eotaxin, HGF, IL-6, IL-13, M-CSF, MCP-3, MMP-3, Osteocalcin, SDF-1a, TNF-R1, TNF-R2, TRAIL and TWEAK (Table 4). The data for Bangladesh and Japanese patients, divided into healthy, mild, and severe cases are shown in Fig. 2. BAFF, CTACK, HGF, IL-6, M-CSF, MMP-3, TNF-R1, and TNF-R2 were higher in the severe group than in the mild group, while TRAIL, osteocalcin, and TWEAK showed a decreasing trend. The patients’ final outcomes and the percentage of those outcomes that were correctly predicted by the model are shown in Table 5.

**Table 4:** The best combination of predictive markers.

	Coefficients
(Intercept)	− 11.615
BAFF	0.663
CTACK	0.215
Eotaxin	0.379
HGF	1.854
IL-6	0.497
IL-13	0.491
M-CSF	0.499
MCP-3	0.029
MMP-3	0.011
Osteocalcin	− 0.595
SDF-1a	− 0.569
TNF-R1	1.048
TNF-R2	1.983
TRAIL	− 1.668
TWEAK	− 0.734

**Table 5:** Percentage of correct predictions for each wave of COVID-19 in Japan by markers extracted from the Bangladesh model.

		Bangladesh	Japan					
	Period	2021/12/25–2022/9/21	2020/6/14–2020/10/9	2020/10/10–2021/2/28	2021/3/1–2021/6/20	2021/6/21–2021/10/17	2021/12/26–2022/6/19	
			2nd wave	3rd wave	4th wave	5th wave	6th wave	Japan
	Main variant	Omicron	Wuhan	Wuhan	Alpha	Delta	Omicron	Total
Final outcome	Prediction	n (%)						
Mild	Mild	53 (41.1)	3(15.8)	12 (16.2)	9 (20.9)	8 (25.0)	6 (20.7)	38
Mild	Severe	14 (10.9)	0(0)	7 (9.5)	2 (4.7)	2 (6.3)	2 (6.9)	13
Severe	Mild	13 (10.1)	5 (26.3)	10 (13.5)	14 (32.6)	3 (9.4)	3 (10.3)	41
Severe	Severe	50 (38.7)	11 (57.9)	45 (60.8)	18 (41.9)	19 (59.4)	18 (62.1)	105
		129	19	74	43	32	29	197
Correct (%)		79.8	73.7	77.0	62.8	84.4	82.8	

### Attempting to predict prognosis in Japanese patients by extracted predictive markers

The Bangladesh training data set (Table 4) were fitted to the data of Japanese patients to predict their prognosis and compare the predictions to patients’ actual clinical outcomes. As shown in Fig. 1B, the results yielded an AUC value of 0.9219 suggesting that the extracted biomarkers made accurate predictions of the prognosis of patients in Japan (Table 5). Comparing the biomarker predictions with the actual clinical outcomes of Japanese subjects, particularly in the second and fourth waves, showed that a high percentage of subjects (26.3% and 39.5%, respectively) were judged as “mild” initially, even though their actual outcome was “severe/moderate”. However, the percentage of correct predictions increased in the 5th and 6th waves. In addition, 60% of the respondents whose predictions were inaccurate were aged 70 years or older, with 40% being over 80 years of age. This suggests that elderly patients are at higher risk of deterioration and should be carefully monitored, even if they initially present with only mild symptoms^23,24^.

In waves 5 and 6, after the vaccine was widely available in Japan, the accuracy rate of the predictive model exceeded 80%. In Bangladesh, approximately 88% of patients were vaccinated, and n Japan, the vaccine became available to the elderly on May 24, 2021, and to ages 12 and older in August. By August, more than 80% of the elderly had been vaccinated. Our results indicate that the predictive biomarker panel is particularly useful in determining the risk of severe disease in COVID-19 patients in environments where public vaccination rates are high.

## Discussion

Since late 2021, the development and widespread use of COVID-19 vaccines and therapeutics has reduced the severity of the disease. As a result, many patients are cured with medication and home care, however, some later become seriously ill and require hospitalization. It is therefore important to determine early in the course of the disease whether patients will recover from the mild symptoms or will worsen and need to be hospitalized. We therefore measured serum cytokine/chemokine/soluble receptor levels in untreated COVID-19 patients presenting at the MILD stage within 7 days of the onset of the disease. The patients were then identified whether they improved as they were or deteriorated to moderate/severe, from which deterioration markers were calculated using the least absolute shrinkage selection operator (LASSO) regression analysis method. We believe that cytokines/chemokines/soluble receptors in the serum reflect a variety of conditions, including the patient's underlying disease, and therefore can be used to determine the worsening status even in the absence of clinical background information.

Data from Evercare Hospital Dhakab in Bangladesh, where patients had nearly identical viral strains, was used as training data for the analysis. From the data 15 biomarkers were extracted: BAFF, CTACK, Eotaxin, HGF, IL-6, IL-13, M-CSF, MCP-3, MMP-3, Osteocalcin, SDF-1a, TNF-R1, TNF-R2, TRAIL and TWEAK (Table 4)^25^. Based on this group of biomarkers, we predicted patient prognosis by applying it to the cytokines/chemokines/soluble receptors data of Japanese patients. The Bangladesh data was collected over 9 months, whereas the Japanese data was collected over a 2-year span. The Japanese data included patients from wave 2 to wave 6 during which time the viral strains were evolving. Therefore, when calculated, the correct response rate from wave 2 to wave 4 was slightly lower, especially in wave 4, where there were more alpha strains (Table 5). This may be because the alpha strain is highly infectious and carries a high risk of mortality.

Interestingly, during waves 2–4, the model tended to underestimate the severity of disease progression in elderly patients, with 60% of the incorrectly predicted patients being over 70 years of age, and 40% being over 80 years old. These cases were initially predicted by the bio-markers to have a mild outcome but patients actually became severe; disease severity was also incorrectly predicted in older patients whose cytokine levels did not significantly increase. Based on these results, it can be said that the biomarkers selected in this study are very effective for predicting the prognosis of COVID-19 patients after widespread vaccination among the public and that caution should be exercised with elderly patients, even if their symptoms are initially classified as mild.

Data from Evercare Hospital Dhakab, Bangladesh, showed that CTACK, G-CSF, HGF, IL-2Ra, IL-6, IL-8, IL-10, IL-12p40, M-CSF, PDGF, SCF, BAFF, CD-30, CD-163, sgp130, IL-11, MMP-3, Pentraxin-3, TNF-R1, TNF-R2 and TSLP were significantly higher in the severe group than in the mild group. The inclusion of many of the markers that differ between severe and mild cases, such as CTACK, HGF, IL-6, M-CSF, BAFF, MMP-3, TNF-R1 and TNF-R2. In this study, differential markers identified for mild and severe COVID-19 cases support the validity of these markers.

As shown in Fig. 2, BAFF, CTACK, HGF, IL-6, M-CSF, MCP-3, MMP-3, TNF-R1 and TNF-R2 tended to increase when patients worsened. We theorize that there may be an underlying mechanism that connects these biomarkers with COVID severity. Among these markers some are associated with inflammation, mortality or lung damage. For example, IL-6, an important mediator of cytokine release syndrome (CRS) toxicity^23,26–34^, signals inflammatory response leading to coagulation, similar to HGF^35^. Additonally, expression levels of HGF and MCP-3 were reported to correlate positively with the Murray score used to assess the severity of lung injury in acute respiratory distress syndrome (ARDS)^14,36–38^. Other cytokines such as MMP3 also play an important role in lung pathological processes such as ARDS, ALI, and lung fibrosis^39–41^. Predictors of motality include soluble tumor necrosis factor receptor 1 and 2 which have been reported as predictive markers of death in patients with severe COVID-19^42,43^. As well, serum TWEAK levels rose during the first week of patients being in intensive care unit (ICU), whereas a decline to baseline values were observed in the second week post-ICU admission (*p* = 0.032) but not for patients who died while in hospital^25^. An analysis of receiver-operator characteristics demonstrated that serum TWEAK at the time patients were admitted to ICU is a significant predictor of in-hospital mortality (AUC = 0.689, *p* = 0.019) in that TWEAK showed a decreasing trend when patients worsened (Fig. 2). Finally BAFF levels hinted at significantly higher risk of both in-hospital and 30-day mortality^44^. To summarize, many of the predictive bio-markers we identified were associated with symptoms typical of COVID-19 in particular lung damage which exacerbates symptoms in COVID patients compared to other viral diseases such as influenza.

While several cytokines and chemokines have been identified as markers for COVD-19 exacerbation, there are still few reports on markers for COVID-19 in patients that have been vaccinated. This study, successfully identified a panel of 15 biomarkers that could predict, with particularly high accuracy, whether vaccinated COVID-19-infected individuals would subsequently have mild symptoms or worsen to medium or severe states that required inpatient treatment. This was done by quantifying serum cytokines within seven days of COVID-19 onset.

A major strength of this study includes the use of a standardized, well-characterized dataset from Bangladesh to develop the predictive model, and the subsequent validation of the model using a diverse dataset from Japan, which spanned multiple COVID-19 variants. The consistent performance of the model across these two distinct populations suggests its robustness. As well, another strength of this predictive model is that blood cytokines and chemokines can be used to predict patient outcome without other clinical markers or details relating to patients’ history of other health issues that may affect the underlying disease status of the patient. Our previous studies have shown that serum cytokine/chemokine levels also reflect the patient's overall health, including the state of any underlying diseases^45,46^ and this allows the model to have potential for broader application. As well, even though high percentages of the population is vaccinated, the future of COVID-19 is not certain, and there are still moderate to severe cases presenting at hospitals in addition to mortality among patients diagnosed as mild cases. Our predictive model could help to alleviate hospital congestion by quickly identifying patients that can recover safely as out-patients, while identifying mild cases that will subsequently worsen so that these patients can be hospitalized and receive preventative treatment. This is important particurly in Japan, since as an aging nation, it has a high percentage of patients over 70 who are vulnerable to deterioration. As well, similar to other least developed nations, public resources have been drained in Bangladesh due to the pandemic, hence being able to ascertain with high accuracy which COVID-19 cases are appropriate for out-patient treatment versus those that require hospitalization can go a long way in using the country’s limited and strained resources efficiently, while still protecting the health of citizens.

## Materials and methods

### Subjects

#### Patients with COVID-19 in Bangladesh

Patients with clinically suspected SARS-CoV-2 infection who visited Evercare Hospital Dhaka between December 25, 2021 and September 21, 2022 were considered for this study. The study was approved by the Ethical Practice Committee of Evercare Hospital Dhaka (approval number ERC 33/2022-01) and the Research Ethics Committee of the Research Institute for Microbial Diseases, Osaka University, Japan (No. 2021-3).

From the patients considered, 129 confirmed COVID-19 patients who were within 7 days of onset were included. Most of these cases in Bangladesh coincide with the sixth and part of the seventh wave in Japan. Patients were recruited at the first visit to the hospital, blood serum was collected, and the attending physician classified the outcomes as mild, moderate, or severe according to the criteria established by the World Health Organization (WHO)^47^ (Table 1), at the time of discharge. Disease severity was not determined on the patients’ first visit, rather it was on their case sheets at the time they were discharged from the hospital. Laboratory confirmed Mild COVID-19 cases were those with one or more symptoms (e.g., fever, cough, runny nose, fatigue, headache, nausea, vomiting, diarrhea, chest pain, abdominal pain, and loss of taste or smell), but lacked shortness of breath, dyspnea on exertion, and abnormal radiological findings. Laboratory confirmed Moderate COVID-19 cases were those with pneumonia, with oxygen saturation > 93% and may have required low oxygen support. Severe cases developed COVID-19 pneumonia and required hospitalization; patients had dyspnea, respiratory frequency ≥ 30 breaths /min, blood oxygen saturation ≤ 93% on room air, lung infiltrates > 50%, and may have required mechanical ventilation and/or ICU support.

All patients visited the hospital with mild symptoms; 64 subsequently remained in mild condition, 46 declined to moderate, and 19 deteriorated to severe stage . The mean time of hospital visit from onset of symptoms was 2.3 ± 0.12 days for mild patients, 2.3 ± 0.14 days for moderate, and 3.0 ± 0.37(mean ± SE) days for severe patients. COVID-19 were diagnosed based on the PCR tests and the date of onset and vaccination status was recorded by the doctor with the patients’ interview. Most of the patients’ laboratory findings were reported preciously (ref.^48^).

#### Patients with COVID-19 in Japan

This sample consisted of 197 patients with clinical suspicion of SARS-CoV-2 infection who were admitted to Habikino hospital, and Tokushukai Hospital from the end of June 2020 to the middle of June 2022^49,50^. All patients provided blood samples on their first visit and written informed consent and the study was approved by the Ethics Committee of Osaka Habikino Medical Center (Approved ID: 150-7), Tokushukai Hospital (TGE01547) and Louis Pasteur Center for Medical Research (LPC.29). This study followed the principles of the Declaration of Helsinki, and was approved by the institutional review board of Osaka University Hospital (No-885). Data for healthy subjects were obtained from Louis Pasteur Center for Medical Research (LPC.8 and LPC.25).

In Japan, the disease severity of patients was determined at hospital admission according to The Guideline for Medical Treatment of COVID-19 (https://www-mhlw-go-jp/content/000785119-pdf). COVID-19 is classified as Mild, Moderate I, Moderate II, and Severe. However, for comparison with Bangladesh, Moderate II and Severe cases were combined and considered as Severe. The severity of illness here refers to the final outcome of the patient and not their condition at the time they were admitted to hospital. The breakdown of patients, the severity classification and age distribution are shown in Table 1. As indicated in Table 1, 197 untreated COVID-19 patients who visited Habikino and Tokushukai Hospitals within 7 days of onset were included. The date of onset was determined by the doctor based on the results of PCR tests and interviews with the patient. The mean number of days to onset were Mild: 3.08 ± 0.32, Moderate: 3.24 ± 0.45, and Severe: 3.71 ± 0.21 days. Ninety one healthy Japanese subjects with an average age of 63.6 ± 1.9 years were also included.

#### Cytokines/chemokines/soluble receptors assay

Cytokines, chemokines, and soluble receptors were quantified using Bio-Plex 200, a multiplex cytokine array system (Bio-Rad Laboratories, CA, USA) according to the manufacturer's instructions. Blood sera from healthy subjects and COVID-19 patients were collected and centrifuged at 1600 g for 10 min. Serum samples were frozen at − 80 °C until they were analyzed. We simultaneously quantified cytokines, chemokines, and soluble receptors. The Bio-Plex Human Cytokine 48-Plex Panel and Inflammation Panel (Bio-Rad Laboratories, CA, USA) was used to simultaneously quantify 78 items: CTACK, Eotaxin, FGF basic, G-CSF, GM-CSF, GRO-α, HGF, IFN-α2, IFN-γ, IL-1α, IL-1β, IL-1ra, IL-2, IL-2Rα, IL-3, IL-4, IL-5, IL-6, IL-7, IL-8, IL-9, IL-10, IL-12(p40), IL-12(p70), IL-13, IL-15, IL-16, IL-17, IL-18, IP-10, LIF, MCP-1(MCAF), MCP-3, M-CSF, MIF, MIG, MIP-1α, MIP-1β, β-NGF, PDGF-BB, RANTES, SCF, SCGF-β, SDF-1α, TNF-α, TNF-β, TRAIL, VEGF) and inflammation panel (37 plex: APRIL, BAFF, CD30, CD163, Chitinase, sgp130, IFN-α 2, IFN-β, IFN-γ, sIL-6Ra, IL-10, IL-11, IL-12(p40), IL-12 (p70), IL-19, IL-20, IL-22, IL-26, IL-27, IL-28A, IL-29, IL-32, IL-34, IL-35, LIGHT, MMP-1, MMP-2, MMP-3, Osteocalcin, Osteopontin, Pentraxin-3, sTNF-R1, sTNF-R2, TSLP, TWEAK. The following were excluded from the inflammation panel for data analysis due to overlap with 48-plex: IFN-α2, IFN-γ, IL-2, IL-8, IL-10, IL-12(p40), and IL-12(p70). Patients’ samples were quantified several times over a 2-year period. Since there were lot-to-lot and measurement-to-measurement errors, these data were corrected based on the values of healthy subjects. The Bangladeshi samples were simultaneously measured with healthy Japanese subjects as a control, and the values of the healthy subjects were used as a reference to correct for lot-to-lot and inter-measurement errors; this was then used to make comparisons with the serum samples of patients in Japan. Biomarkers with large inter-kit errors and low measurement sensitivity were excluded from the analysis: IL-19, IL-20, IL-26, IL-28A, IL-29, IL-35, LIGHT.

### Statistical analysis

The distribution of cytokine/chemokine/soluble receptor values in healthy controls was analyzed to determine whether the raw values or log-transformed values were more normally distributed. All parameters had log-transformed values that were more normally distributed (data not shown), and so these were used in our analysis^45^. The t-test results for the data used in Bangladesh and Japan, categorized by mild, moderate, and severe disease are shown in Fig. 2. To correct for inter-measurement error, Japanese healthy controls were used as reference and adjusted accordingly. To allow for multiple comparisons, the *p*-values were corrected by the Holm using the p.adjust function in the stats package of the R language.

ANOVA was performed and quantitative data was presented as means ± SEM. The significance of the difference between the groups was evaluated using Dunnett’s test with a value of *p* < 0.05 considered significant. All statistical analyses were carried out with JMP 20.0 Statistical Software (JMP Statistical Discovery LLC, NC, USA).

The aim of the study was to use cytokines/chemokines/soluble receptors data, collected within 7 days of COVID onset, to predict whether a patient would subsequently deteriorate to a moderate disease state or worse and require hospitalization. To achieve this, we employed a binary logistic regression model. To refine the predictors, we utilized the Least Absolute Shrinkage and Selection Operator (LASSO) regression to select relevant cytokines as candidate markers^22^. The selection of the optimal number of variables was guided by Leave-One-Out Cross Validation (LOO CV). LOO-CV is known to be over-trained, however, in this case, the correct generalization performance could be evaluated using validation data, without the need to consider variations due to partitioning (such as k-fold).

Due to the disproportionate distribution of disease severities in the Bangladeshi COVID-19 patients, we applied weights to the Logistic LASSO regression model to minimize potential bias in the data. In line with the WHO severity classification, the groups that did not require hospitalization were defined as those with mild illness and recovered, while the groups that were hospitalization and required medical treatment were defined as moderate and severe. In addition, healthy individuals were added to the “no hospitalization group” so that the model would correctly predict that hospitalization was not necessary. Here, the number of persons in each group is clinically known. Characteristic markers for the smallest group, especially for the most severely ill patients, may be buried. Therefore, we determined the ratio of the number of people in each breakdown and weighted the objective variable by its reciprocal (the glmnet function of the glmnet package has a weight argument, which can be used to assign weights to the objective variable).

The performance of the logistic regression model was assessed using both training and validation datasets. This included determining the Area Under the Curve (AUC) from the Receiver Operating Characteristic (ROC) curve, and calculating performance metrics such as specificity and sensitivity. All analyses were conducted using the R language v4.2 (https://www.r-project.org/), with the glmnet 4.1-7 package supporting variable selection and logistic regression analysis. The pROC package version 1.18.4 was utilized to evaluate the model's performance through the ROC curve and AUC.

## Data Availability

The data that support the findings of this study are available from Osaka Habikino Medical Center, Tokushukai Hospital and Evercare Hospital Dhaka. However, the data is not publicly available since it was obtained under license for the current study. Upon reasonable request howoever, the data may be obtained from the corresponding author Kazuko Uno with permission from the respective institutions.
